# Breast Cancer Subtype is Associated With Axillary Lymph Node Metastasis: A Retrospective Cohort Study

**DOI:** 10.1097/MD.0000000000002213

**Published:** 2015-12-07

**Authors:** Zhen-Yu He, San-Gang Wu, Qi Yang, Jia-Yuan Sun, Feng-Yan Li, Qin Lin, Huan-Xin Lin

**Affiliations:** From the Department of Radiation Oncology, Sun Yat-sen University Cancer Center, State Key Laboratory of Oncology in South China, Collaborative Innovation Center for Cancer Medicine, Guangzhou (Z-YH, J-YS, F-YL, H-XL); Department of Radiation Oncology, Xiamen Cancer Center, the First Affiliated Hospital of Xiamen University, Xiamen (S-GW, QL); and Department of Nasopharyngeal Carcinoma, Sun Yat-sen University Cancer Center, State Key Laboratory of Oncology in South China, Collaborative Innovation Center of Cancer Medicine, Guangzhou, People's Republic of China (QY).

## Abstract

The purpose of this study was to assess whether breast cancer subtype (BCS) as determined by estrogen receptor, progesterone receptor, and human epidermal growth factor receptor 2 can predict the axillary lymph node metastasis in breast cancer.

Patients who received breast conserving surgery or mastectomy and axillary lymph node dissection were identified from 2 cancer centers. The associations between clinicopathological variables and axillary lymph node involvement were evaluated in univariate and multivariate regression analyses.

A total of 3471 patients met the inclusion criteria, and 53.0% had axillary lymph node metastases at diagnosis. Patients with hormone receptor (HR)−/human epidermal growth factor receptor 2 (HER2)− subtype had a higher grade disease and the lowest rate of lymphovascular invasion. Univariate and multivariable logistic regression analyses showed that BCS was significantly associated with lymph node involvement. Patients with the HR−/HER2− subtype had the lowest odds of having nodal positivity than those with other BCSs. HR+/HER2− (odds ratio [OR] 1.651, 95% confidence interval [CI]: 1.349–2.021, *P* < 0.001), HR+/HER2+ (OR 1.958, 95%CI 1.542–2.486, *P* < 0.001), and HR−/HER2+ (OR 1.525, 95%CI 1.181–1.970, *P* < 0.001) tumors had higher risk of nodal positivity than the HR−/HER2− subtype. The other independent predictors of nodal metastases included tumor size, tumor grade, and lymphovascular invasion.

Breast cancer subtype can predict the presence of axillary lymph node metastasis in breast cancer. HR−/HER2− is associated with a reduced risk of axillary lymph node metastasis compared to other BCSs. Our findings may play an important role in guiding axillary treatment considerations if further confirmed in larger sample size studies.

## INTRODUCTION

Axillary lymph node status is an important factor in determining the staging, prognosis, and treatment of breast cancer patients.^1^ However, there is still a great deal of controversy exists regarding the management of axillary lymph nodes in breast cancer. Sentinel lymph nodes have been used in the evaluation of axillary lymph node status in early stage breast cancer patients.^2,3^ Several predict factors of axillary lymph node metastasis have been described such as age, tumor location, tumor size, histologic grade, and lymphovascular invasion (LVI).^4–8^

Recently, the immunohistochemical analyses of expression of estrogen receptor (ER), progesterone receptor (PR), and human epidermal growth factor receptor 2 (HER2), are widely used to divide into 4 breast cancer subtypes (BCSs). The BCSs are used to guide the system treatment, and predict response to therapy and disease outcome.^9–13^ However, the role of BCS on axillary lymph node status has not been well established.^14–18^ In this study, we retrospectively reviewed the clinicopathological data of breast cancer patients from 2 cancer centers aiming to investigate the association between the BCS status and axillary lymph node involvement, and to assess whether the BCS might be used to guide axillary management decisions.

## PATIENTS AND METHODS

### Patients

Clinicopathological information of patients who were treated at the First Hospital of Xiamen University (Xiamen Cancer Center, XMCC) between January 2008 and March 2012 and Sun Yat-sen University Cancer Center (SYSUCC) between January 1998 and December 2007 were collected. The inclusion criteria were: (1) female patients with unilateral invasive breast cancer without distant metastasis at diagnosis; (2) received mastectomy or breast-conserving surgery and axillary lymph node dissection; (3) without neoadjuvant therapy; (4) had complete clinicopathological information including ER, PR, and HER2 status. The study was approved by the ethics committee of the First Affiliated Hospital of Xiamen University and SYSUCC.

### Classification Criteria for Patients

BCSs were defined as follows: HR+/HER2− (ER+ and/or PR+, HER2−), HR+/HER2+ (ER+ and/or PR+, HER2+), HR−/HER2+ (ER−, PR−, and HER2+), and HR−/HER2− (ER−, PR−, and HER2−, TNBC). Hormone receptor (HR) positivity was defined as >1% of ER or PR positive cells by immunohistochemistry. HER2 positivity was defined as immunohistochemical grade of 3+ before 2003 and was defined as immunohistochemical grade of 3+, or 2+ was determined by fluorescence *in situ* hybridization (FISH) after 2003. The cutoff point for Ki-67 was 25% according to our previous reports.^19^

Nodal metastasis was defined as the presence of any tumor cells in a lymph node. Breast cancer was staged according to the seventh edition American Joint Committee on Cancer (AJCC)/ Union for International Cancer Control (UICC) staging system. Clinicopathological factors that would impact the axillary lymph node status were then analyzed, including age, menstrual status, tumor size, histologic grade, LVI, ER, PR, HER2, Ki-67, and BCS.

### Statistical Analysis

All data were analyzed using the SPSS statistical software package (version 16.0; IBM Corporation, Armonk, NY). The χ^2^ test and Fisher's exact probability tests were used for categorical variables, and analysis of variance for continuous variables, to compare the distribution of clinicopathological characteristics among BCSs. The relationship between patient characteristics and axillary lymph node metastases was examined by univariate and multivariable logistic regression analyses. Factors that were statistically significant in univariate analysis were entered into multivariable logistic regression analysis. A *P* value < 0.05 was considered significant in all analyses.

## RESULTS

A total of 3471 patients included in this study, 662 (19.1%) patients were from XMCC and 2809 (80.9%) patients were from SYSUCC. Patient and tumor characteristics are shown in Table 1. There were 53.0% of patients had nodal positivity, 57.9% of patients were ER+, 63.4% were PR+, and 33.1% were HER2+. Patients’ tumors were categorized as HR+/HER2− in 1763 (50.8%), HR+/HER2+ in 666 (19.2%), HR−/HER2+ in 481 (13.9%), and HR−/HER2− in 561 (16.1%). Patient and tumor characteristics categorized by BCS are shown in Table 2. Among the BCSs, there were significant differences in age (*P* = 0.021), menopausal status (*P* < 0.001), tumor size (*P* < 0.001), nodal stage (*P* < 0.001), grade (*P* < 0.001), LVI (*P* = 0.005), and Ki-67 (*P* < 0.001). Patients with HR−/HER2− subtype had a higher grade disease and the lowest rate of LVI. Significantly more patients with high Ki-67 expression were HR+/HER2− subtype than did those with other BCSs (*P* < 0.001).

**TABLE 1:**
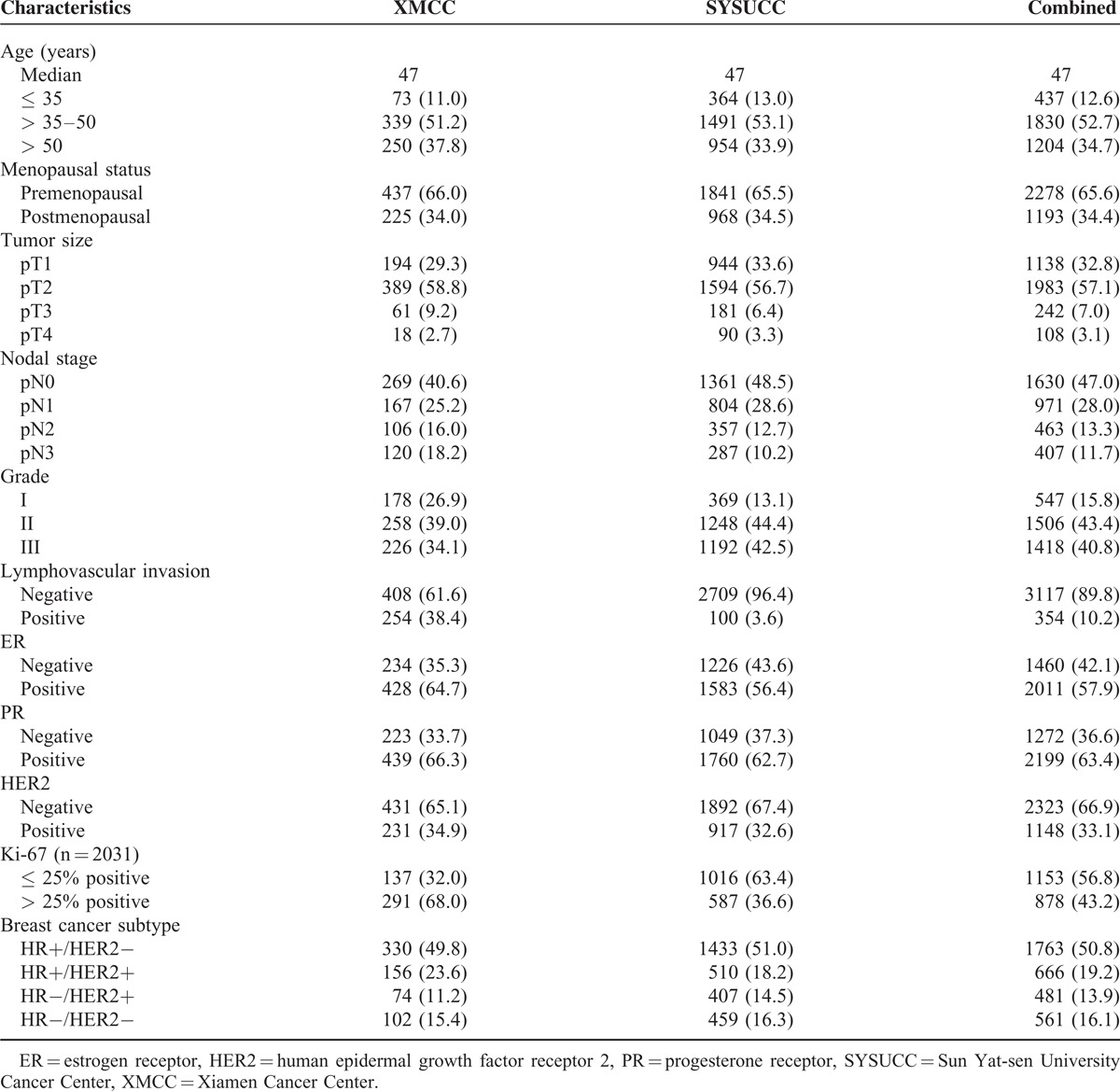
Clinicopathological Characteristics of Patients With Breast Cancer in 2 Cancer Center

**TABLE 2:**
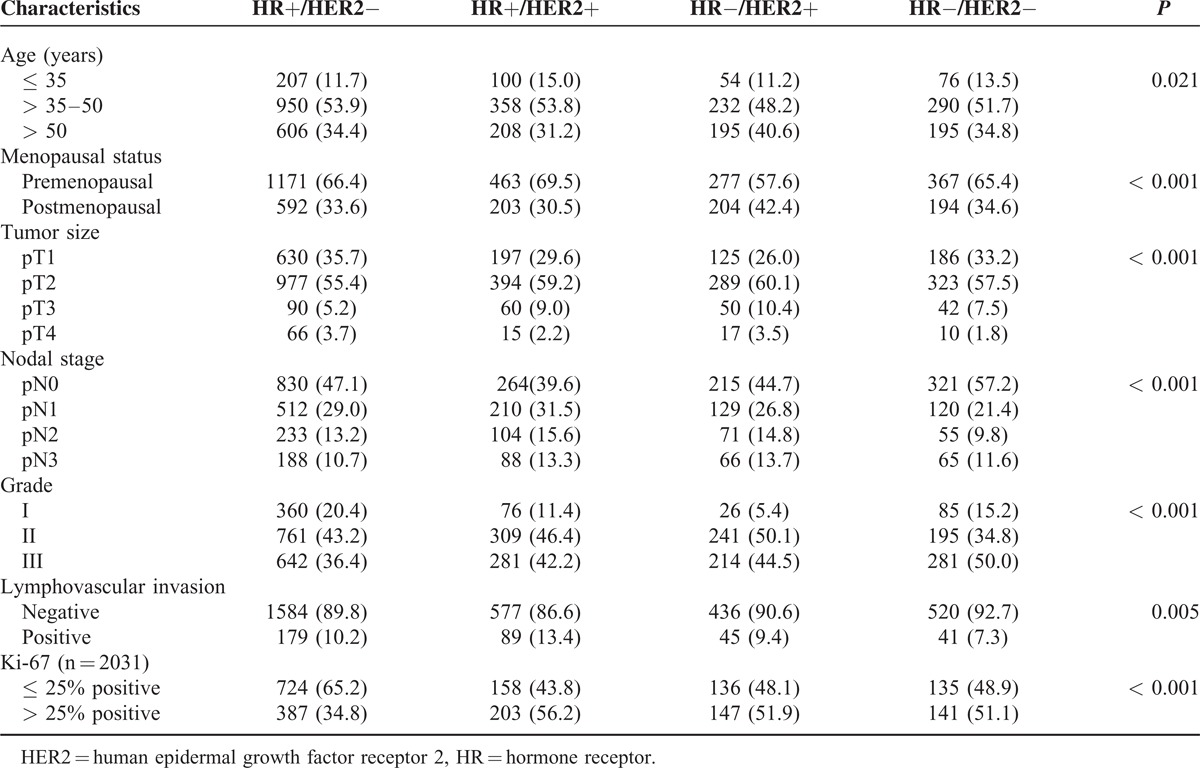
Patient and Tumor Characteristics by Breast Cancer Subtype

**FIGURE 1:**
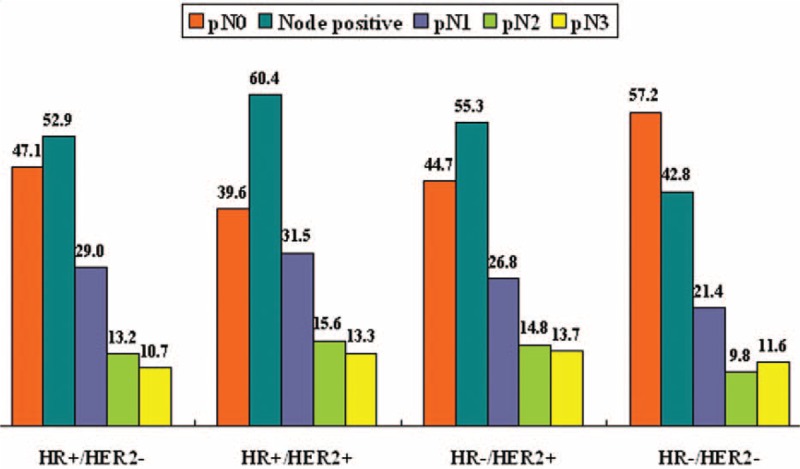
The frequency (%) of nodal positivity according to the breast cancer subtype.

The results of the univariate logistic regression analysis are shown in Table 3. Larger tumor size, higher grade, LVI, ER+, PR+, and HER2+ were associated with a higher risk of lymph node metastases. Breast cancer subtype was a significant predictor of nodal positivity. The HR+/HER2−, HR+/HER2+, and HR−/HER2+ subtypes had a significantly higher risk of having positive lymph nodes, whereas the HR−/HER− subtype was associated with a reduced risk of lymph node metastasis (Fig. 1).

**TABLE 3:**
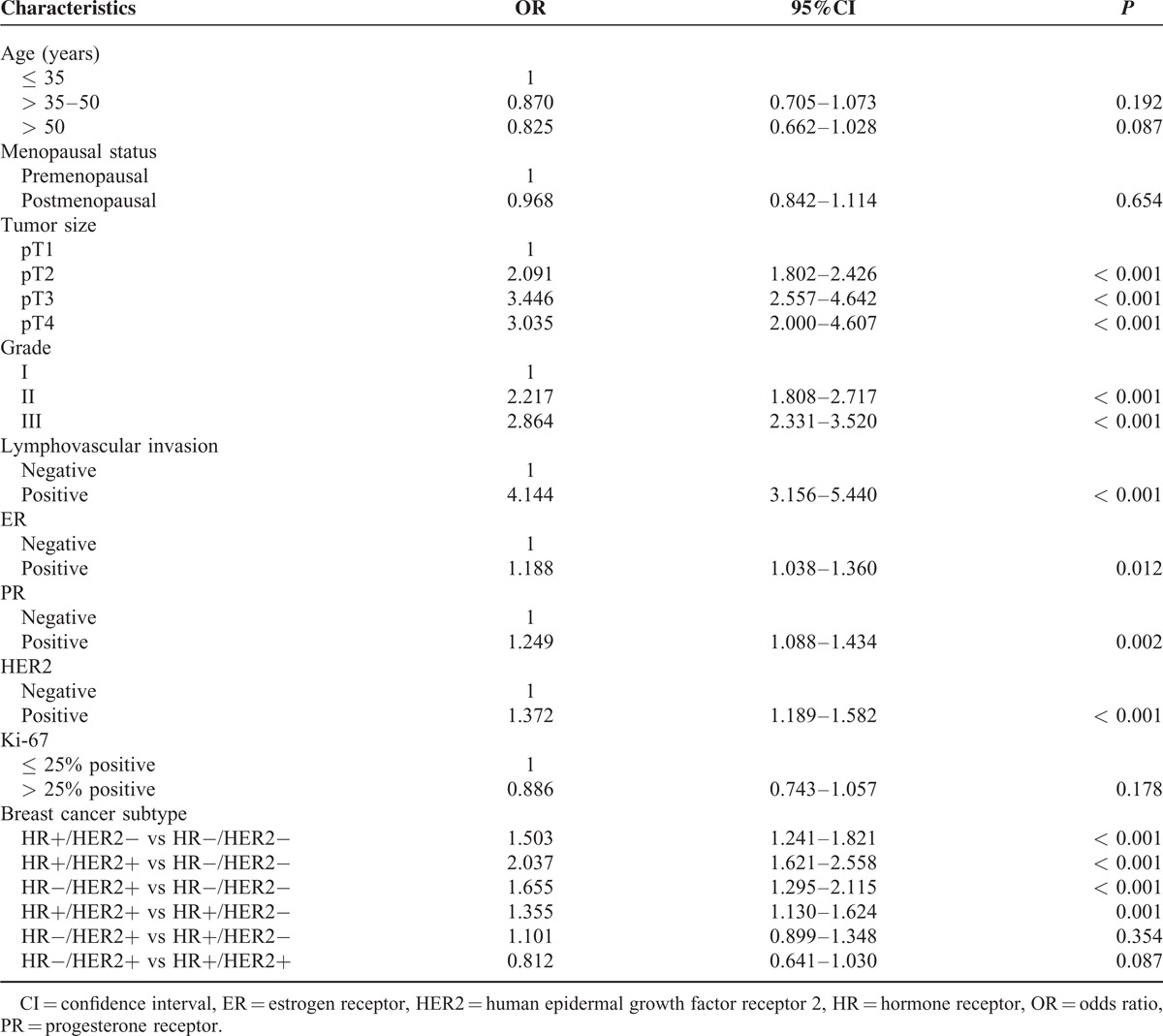
Univariate Logistic Regression of Axillary Lymph Node Metastasis

The results of the multivariable analysis are shown in Table 4. In model 1, when adjusted for tumor size, grade, LVI, ER, PR, and HER2, larger tumor size, higher grade, LVI, PR+, and HER2+ were risk factors of axillary lymph nodes metastasis. In model 2, after adjusting for tumor size, grade, LVI, and BCS, tumor size, grade, and LVI remained predictors of lymph node metastases. Patients with the HR−/HER2− subtype had the lowest odds of nodal positivity compared to other BCSs. HR+/HER2− (odds ratio [OR] 1.651, 95% confidence interval [CI]: 1.349–2.021, *P* < 0.001), HR+/HER2+ (OR 1.958, 95%CI 1.542–2.486, *P* < 0.001), and HR−/HER2+ (OR 1.525, 95%CI 1.181–1.970, *P* < 0.001) tumors had higher risk of have lymph node metastases than the HR–/HER2– subtype. The HR−/HER2+ subtype had a reduced risk of axillary lymph node metastasis as compared to the HR+/HER2+ subtype, with an OR of 0.779 (95 % CI: 0.609–0.997, *P* = 0.048).

**TABLE 4:**
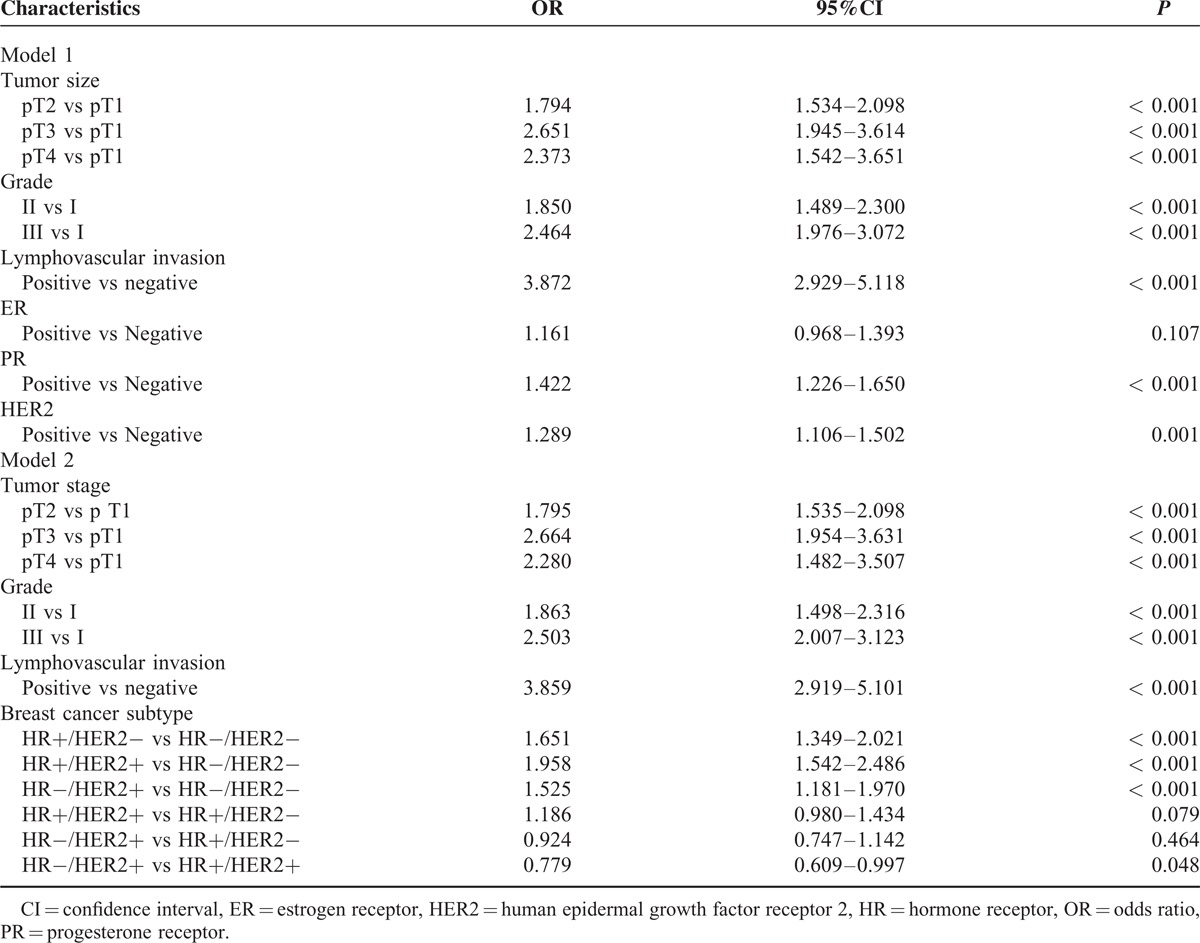
Multivariate Logistic Regression of Axillary Lymph Node Metastasis

## DISCUSSION

In the present study, we assessed the clinical value of BCS for predicting axillary lymph node metastasis in breast cancer patients and the results showed that HR−/HER2− subtype was associated with a lower risk of lymph node metastasis as compared to other BCSs.

The status of the axillary lymph node is an important prognostic factor in breast cancer patients. Studies from Western countries showed that age, tumor location, tumor stage, grade, and LVI could be used to evaluate the axillary lymph node status.^4–8^ Our findings from a Chinese population showed that tumor stage, grade, and LVI were also factors affecting the axillary lymph node status, but no relationship with age. Similar findings were also observed in a study conducted in Korea.^16^ There is evidence showing that women are more likely to have positive lymph nodes with increasing age, but the age distribution of breast cancer patients in Eastern was different from those of Western countries.^20,21^ Thus, the value of age as a predictor of axillary lymph node status is influenced by other factors.^22^

In this study, 3471 patients were included from 2 cancer centers and the results showed that TNBC patients had a lower risk of axillary lymph node metastasis as compared to other BCS patients. The finding of this study was similar to the results of previous studies.^14,15,23,24^ A study of the Danish Breast Cancer Cooperative Group database that included 20,009 patients showed that TNBC patients had a reduced risk of axillary lymph node involvement than other BCSs when adjusted for other risk factors.^14^ Ugras et al^15^ investigated 11,596 patients with breast cancer and found that nodal metastases were least frequent in TNBC as compared with other subtypes. A Chinese National Cancer Center study that included 3,198 patients showed that the probability of positive lymph nodes in TNBC patients was significantly lower than that in patients with other subtypes (28.2% vs 43.3–44.8%), although without multivariate analysis.^23^ In a Surveillance, Epidemiology, and End Results study with 7,274 patients, the HR+/HER2– subtype had a higher rate of lymph node metastasis at diagnosis than the TNBC.^24^ However, the value of BCS for predicting axillary lymph node status is still controversial. TNBC patients had a higher risk of nodal positivity (OR 2.09) in a Korean study.^16^ In addition, Gangi et al^17^ investigated 2,967 patients and multivariate analysis failed to show a significant difference in the lymph node status among patients with 4 BCSs. Furthermore, Wiechmann et al^18^ reviewed the records of 6,042 patients and reported that TNBC tumors did not have involved lymph nodes more often than non-TNBC. Sample size variation across studies may produce disparate findings in the above studies.

Lymphovascular invasion has been found to be a risk factor for locoregional and distant recurrence in breast cancer patients.^25,26^ Lymphovascular invasion is an obligatory step in tumor metastasis, and therefore may be a surrogate marker for metastatic potential.^27^ However, therapeutic failure is frequently found at 3 to 5 years in TNBC patients with hematogenous metastasis.^28^ Our results showed the frequency of LVI in TNBC patients (7.3%) was statistically lower than that in patients with other BCSs (9.4–13.4%). Ugras et al^15^ also found that the risk for LVI in other BCSs (OR 1.7–2.5) was statistically higher than in TNBC. Based on these findings, we speculate that TNBC patients have a lower risk for axillary lymph node metastasis and might be susceptible to hematogenous metastasis, but not directly associated with lymphatic spread.

Sentinel lymph node biopsy is an important treatment for early breast cancer and is helpful to improve quality of life.^29^ However, a method to accurately evaluate axillary lymph node status is an important prerequisite for sentinel lymph node biopsy. In patients with positive sentinel lymph nodes, the risk for positive nonsentinel nodes in TNBC patients is significantly lower than in Luminal A and Luminal B patients, but similar to that in HER2 overexpressing patients.^30^ Freedman et al^31^ found that TNBC had the lowest risk of nonsentinel lymph node metastasis in breast cancer patients with positive sentinel lymph nodes as compared to other subtypes. This indicates that BCS may be an important factor determining the need for axillary lymph node dissection in patients with breast cancer, and dissection may not be necessary in some TNBC patients. However, breast cancer was not subtyped in the ACOSOG Z0011 clinical trial,^2,3^ and more studies are required to confirm our findings.

There are limitations in our study. First, it is a retrospective study and hence is subject to inherent biases. However, the patients included in this study were from 2 cancer centers, and the results of this study had potential impact of axillary lymph node management decisions in clinical practice. Second, because of the patients period spanned with >10 years, BCS were not determined according to the criteria developed in the St. Gallen International Breast Cancer Conference because some patients did not have immunohistochemistry for Ki-67.^32^ We have not found the value of Ki-67 in predicting lymph node metastases; this confirms findings from previous studies.^33–35^ In addition, the ER, PR, and HER2 expressions were mainly detected by immunohistochemistry, which may bias the results, but the results of immunohistochemistry have been widely used in the treatment option for patients with breast cancer.

## CONCLUSIONS

In conclusion, our results show that BCS as determined by ER, PR, and HER2 status can predict axillary lymph node metastasis in breast cancer. Although TNBC is more aggressive, a lower risk for axillary lymph node metastasis compared to patients with other BCSs. The findings suggest that lymphatic metastasis is not a major pattern of metastasis in HR−/HER2− patients. Our findings may play an important role in guiding axillary treatment considerations if further confirmed in larger sample size studies.
